# Immune checkpoint inhibitors in high‐grade upper tract urothelial carcinoma: Paradigm shift emphasizing organ preservation

**DOI:** 10.1002/bco2.335

**Published:** 2024-03-03

**Authors:** Mahmoud Khalil, Andrew Fishman, Anna Komorowski, Israel Franco, Michael Grasso

**Affiliations:** ^1^ Department of Urology Phelps Hospital, Northwell Health Sleepy Hollow New York USA; ^2^ Department of Urology Ain Shams University Cairo Egypt; ^3^ Department of Hematology and Medical Oncology Phelps Hospital, Northwell Health Sleepy Hollow New York USA; ^4^ Department of Urology Yale School of Medicine New Haven Connecticut USA; ^5^ Department of Urology New York Medical College Valhalla New York USA

**Keywords:** high grade, immune checkpoint inhibitors, immunotherapy, renal urothelial carcinoma, upper tract urothelial carcinoma, ureteral carcinoma

## Abstract

**Objective:**

The aim was to evaluate the role of immune check point inhibitors (ICIs) in patients with high‐grade upper tract urothelial carcinoma (UTUC) who are managed endoscopically when nephroureterectomy (NU) is not feasible, such as in patients who are either not candidates for NU or decline extirpative surgery.

**Methods:**

All patients diagnosed with high‐grade UTUC and managed endoscopically between January 1996 and August 2022 were included in the study. Subsequently, patients were categorised based on their use of ICIs into group 1 (patients who did not receive ICIs) and group 2 (patients who received ICIs). Survival outcomes were assessed using Kaplan–Meier analysis, while a multivariable regression model was employed to analyse the impact of clinical characteristics on survival.

**Results:**

A total of 29 patients were enrolled, with 14 in group 1 and 15 in group 2. Both groups exhibited similar demographic and disease characteristics, including multifocality, laterality and initial tumour size. The median follow‐up period was 29.2 months. Notably, group 2 demonstrated significantly enhanced overall and metastasis‐free survival rates compared to group 1. At 47.8 months, the overall survival rate was 0% (all patients died) in group 1, whereas it was 85.7% in group 2. Similarly, the metastasis‐free survival rate was 0% (all patients had metastatic disease) in group 1 at 40.6 months, whereas it reached 78.0% in group 2. The multivariable analysis indicated a correlation between ICI usage and improved survival outcomes, with a hazard ratio of 0.002.

**Conclusion:**

Utilisation of adjuvant ICIs in the setting of endoscopically treated patients with high‐grade UTUC is associated with significantly improved survival rates. ICIs should be considered in this patient population, however, more studies with larger sample size are warranted.

## 1 INTRODUCTION

Upper tract urothelial carcinoma (UTUC) is a relatively rare disease and can be challenging to treat in advanced and metastatic conditions.^1^ The differentiation of tumour grade plays a pivotal role in formulating an effective treatment approach for UTUC. Radical nephroureterectomy (NU) along with bladder‐cuff excision has traditionally been the standard treatment for high‐risk localised disease. However, studies have demonstrated that low‐grade tumours can be managed ureteroscopically with long‐term success provided a strict surveillance protocol is followed.^2^, ^3^

In case of high‐grade tumours, extirpative surgery is considered the primary treatment, often accompanied by platinum‐based chemotherapy to optimise oncological outcomes.^4^ There remains a subset of patients who are unfit for NU due to multiple comorbidities. This category also includes patients with compelling reasons to preserve renal function, such as those with a solitary kidney, bilateral disease or compromised renal function, where surgical resection could lead to end‐stage kidney disease and the need for haemodialysis. Additionally, some patients may decline extirpative surgery altogether.

While local control of the tumour can be achieved ureteroscopically, this approach is associated with an increased risk of recurrence and progression to invasive disease.^1^ Consequently, this approach is considered non‐curative, and the likelihood of achieving disease‐free status in these selected patients remains uncertain. In a systematic review, endoscopic management of high‐grade UTUC was associated with unfavourable oncological outcomes and diminished 5‐year disease‐specific survival.^5^

Immune checkpoint inhibitors (ICIs)‐based therapy has been extensively studied, and the results have been promising as an adjuvant treatment for urothelial carcinoma.^6^ However, data on utilising systemic ICIs for high‐grade UTUC to improve clinical and survival outcomes remain limited. In this study, we aim to assess the role of ICIs in patients with high‐grade UTUC who were treated endoscopically when NU was not feasible.

## 2 PATIENTS AND METHODS

### 2.1 Study design and patient selection

This is a longitudinal retrospective study in which we queried our maintained institutional database to identify all patients with high‐grade UTUC who were treated endoscopically from January 1996 to August 2022. These patients were subsequently categorised into two groups based on the utilisation of adjuvant ICIs: group 1 (comprising patients who did not receive ICIs) and group 2 (comprising patients who received ICIs). All procedures were performed by a single surgeon (MG). Demographic information, perioperative data and postoperative outcomes were collected. This study was approved by the Institutional Review Board of our centre. All participants provided informed consent for inclusion of relevant health information in the study.

### 2.2 Initial workup and surgical technique

The diagnostic evaluation of patients included computed tomography (CT) urogram or magnetic resonance imaging (MRI) of the abdomen and pelvis, along with additional imaging as part of a metastatic evaluation such as a CT chest and urine cytology. In all patients, diagnostic flexible ureteroscopic mapping of the upper urinary tract was carried out using the previously described ‘no‐touch’ technique.^7^ Representative biopsies were obtained for histopathology using a flat wire basket, along with barbotage specimens for cytopathology. Ablation of visible tumour was performed using laser energy or electrocautery. For large lesions, staged URS was conducted if deemed necessary.

After establishing diagnosis of high‐grade UTUC, and when NU is not feasible, patients were placed on strict surveillance protocol as well as on an appropriate ICI agent after consultation with medical oncology. The surveillance protocol involved ureteroscopic evaluation every 3 months during the first year after initial treatment, and semi‐annually thereafter in case of negative evaluations.

The radiographic follow‐up involved performing a CT urogram or MRI of the abdomen and pelvis biannually in the initial year, followed by annual assessments if initial evaluations were negative. Additionally, metastatic evaluations, either via an additional chest CT scan or a PET/CT (positron emission tomography) scan, were performed on an annual basis.

### 2.3 Data collection

Demographic and clinical data collected were age, sex, initial and last creatinine levels, disease laterality and multifocality, concurrent bladder cancer, prior history of bladder cancer or cystectomy, prior UTUC history, upper tract tumour grade at diagnosis, initial tumour size, follow‐up duration and the specific ICI agent administered to group 2 patients. Additional data collected involved the overall count of ureteroscopies, occurrences of upper tract and bladder recurrence, instances of progression to renal failure, rates of metastasis, incidents of salvage NU and mortality.

### 2.4 Statistical analysis

Descriptive statistics for patient demographics and disease characteristics were computed for the entire sample and for the two treatment groups. Comparison of the patient characteristics between the two treatment groups was carried out using t‐tests (for continuous variables) and Fisher's exact test (for categorical variables). Kaplan–Meier survival estimates were provided for both overall and metastasis‐free survival. Additionally, survival rates at the 12‐month and 24‐month follow‐up points were documented. Bivariate and multivariable Cox proportional hazard regressions were conducted for the overall survival outcome, with ICI use status as the primary exposure of interest. Age, sex, solitary kidney, bilateral disease, multifocality, concomitant bladder cancer and initial tumour size were adjusted for in the multivariable model. All analyses utilised a threshold of *p* < 0.05 for determining statistical significance. The data analyses were performed using SAS 9.4.

## 3 RESULTS

### 3.1 Patient baseline characteristics

Over the study period, a total of 394 patients with UTUC were identified, averaging approximately 15 patients per year. Among these, 205 patients (52%) presented with low‐grade disease and underwent ureteroscopic treatment, while 160 patients (40.6%) had high‐grade disease and underwent nephroureterectomy. Finally, 29 patients (7.3%) had high‐grade disease were treated ureteroscopically and were included in this study.

Within this group of 29 patients, 14 did not receive ICIs (group 1), while 15 received ICIs (group 2). As demonstrated in Table 1, there were no significant differences between the two groups in terms of median age, sex and disease characteristics, including multifocality, laterality, history of bladder cancer, concomitant bladder cancer and initial tumour size.

**TABLE 1:** Demographic and clinical characteristics of the sample.

Patient characteristics	Total sample (*n* = 29); *n* (%)	Group 1/no immune checkpoint inhibitors (*n* = 14); *n* (%)	Group 2/immune checkpoint inhibitors (*n* = 15); *n* (%)	*p*‐value
Age: median (IQR)	75.0 (70.0–79.0)	74.5 (71.0–79.0)	76.0 (65.0–84.0)	0.647^a^
Sex				0.682
Male	21 (72.4)	11 (78.6)	10 (66.7)	
Female	8 (27.8)	3 (21.4)	5 (33.3)	
Initial/last creatinine concentration, mg/dL: mean (SD)	1.7 (0.9)/2.2 (1.4)	2.0 (1.1)/2.5 (1.8)	1.5 (0.6)/2.0 (0.9)	0.207^a^/0.455^a^
Solitary kidney	4 (13.8)	1 (7.1)	3 (20.0)	0.598
Bilateral disease	8 (27.6)	5 (35.7)	3 (20.0)	0.427
Multifocality	16 (55.2)	9 (64.3)	7 (46.7)	0.462
History of bladder cancer	17 (58.6)	10 (71.4)	7 (46.7)	0.264
High grade	12 (41.4)	7 (50.0)	5 (33.3)	
Low grade	6 (20.7)	4 (28.6)	2 (13.3)	
Concomitant bladder cancer	7 (24.1)	3 (21.4)	4 (26.7)	1.000
Cystectomy	5 (17.2)	4 (28.6)	1 (6.7)	0.169
History of upper tract transitional cell carcinoma	16 (55.2)	7 (50.0)	9 (60.0)	0.715
High grade	8 (27.6)	4 (28.6)	4 (26.7)	
Low grade	8 (27.6)	3 (21.4)	5 (33.3)	
Initial tumour size				0.402
Small (<1 cm)	8 (27.8)	5 (35.7)	3 (20.0)	
Medium (1–3 cm)	8 (27.8)	2 (14.3)	6 (40.0)	
Large (>3 cm)	13 (44.8)	7 (50.0)	6 (40.0)	
Follow‐up months: median (IQR)	29.2 (17.9–46.0)	20.2 (17.9–39.4)	41.0 (17.0–72.0)	0.032^a^

Abbreviation: IQR, interquartile range.

^a^ *p*‐values were estimated using *t*‐test. For other variables, *p*‐values were estimated using Fisher's exact test.

In both groups, the indications for conservative ureteroscopic treatment included four (13.8%) patients with a solitary kidney, eight (27.6%) had bilateral disease, 12 (41.3%) with chronic kidney disease, three (10.3%) were not candidates for surgery, and two (6.8%) declined the extirpative surgery. Multifocal disease was recorded in 55.2% of all patients. History of bladder cancer and history of UTUC prior to the initial presentation was recorded in 58.6% and 55.2%, respectively. Concomitant bladder cancer was identified in 24.1% in both groups. Tumour size of greater than 3 cm was the most prevalent (44.8%) among both groups. Two patients in group 1 and one patient in group 2 presented with metastatic disease either regional or distant at the time of initial diagnosis. The remaining patients in both groups had localised disease. Notably, the median follow‐up period was significantly longer in group 1 compared to group 2 (41.0 months vs. 20.2 months, respectively, *p* = 0.032).

### 3.2 Surgical and checkpoint inhibitors data

The average frequency of ureteroscopic treatment in group 1 was 5.8 (range: 3–12) versus 10.0 (range: 1–29) in group 2. Staged initial ureteroscopic treatment of tumour was required in eight patients (57%) in group 1 and in nine (60%) in group 2. UTUC recurrence was reported in all patients in group 1 patients in an average of 2.6 months, while in group 2, recurrence was reported in 67% of patients in an average of 2.7 months.

In each group, three patients developed a new bladder carcinoma after the initial UTUC diagnosis, accounting for 21.4% in group 1 and 20% in group 2. Additionally, both groups experienced bladder recurrences after an average of 3.6 months from the initial diagnosis, all of which were identified during the second or third ureteroscopic evaluation. Chronic kidney disease that eventually progressed to end‐stage renal disease occurred in four patients (28.5%) in group 1, as opposed to one patient (6.7%) in group 2. PD‐L1 expression testing was performed in three patients in group 2, with two of them exhibiting levels greater than 10%.

Patients identified in and after year 2010 were those who received ICIs. The primary ICI used in group 2 was pembrolizumab, which was administered to 80% of the patients. Another ICI utilised was atezolizumab. In group 2, patients received ICIs immediately after a diagnosis of high‐grade UTUC was confirmed by ureteroscopic evaluation and completion of the metastatic workup. The initiation of ICIs was independent of the metastatic status of the disease.

Adverse events related to pembrolizumab were documented in four patients (27%), including colitis, pancreatitis, hypophysitis and interstitial nephritis with associated renal insufficiency. In these cases, pembrolizumab was substituted with either atezolizumab, nivolumab, durvalumab or erdafitinib. In most cases, a brief course of high‐dose corticosteroids (such as prednisone at a dosage of 0.5–1 mg/kg daily) could be administered as a first‐line treatment.

### 3.3 Survival rates and multivariate analysis

Regional and distant metastasis were observed in 92.8% and 20% in groups 1 and 2, respectively.

**FIGURE 1:**
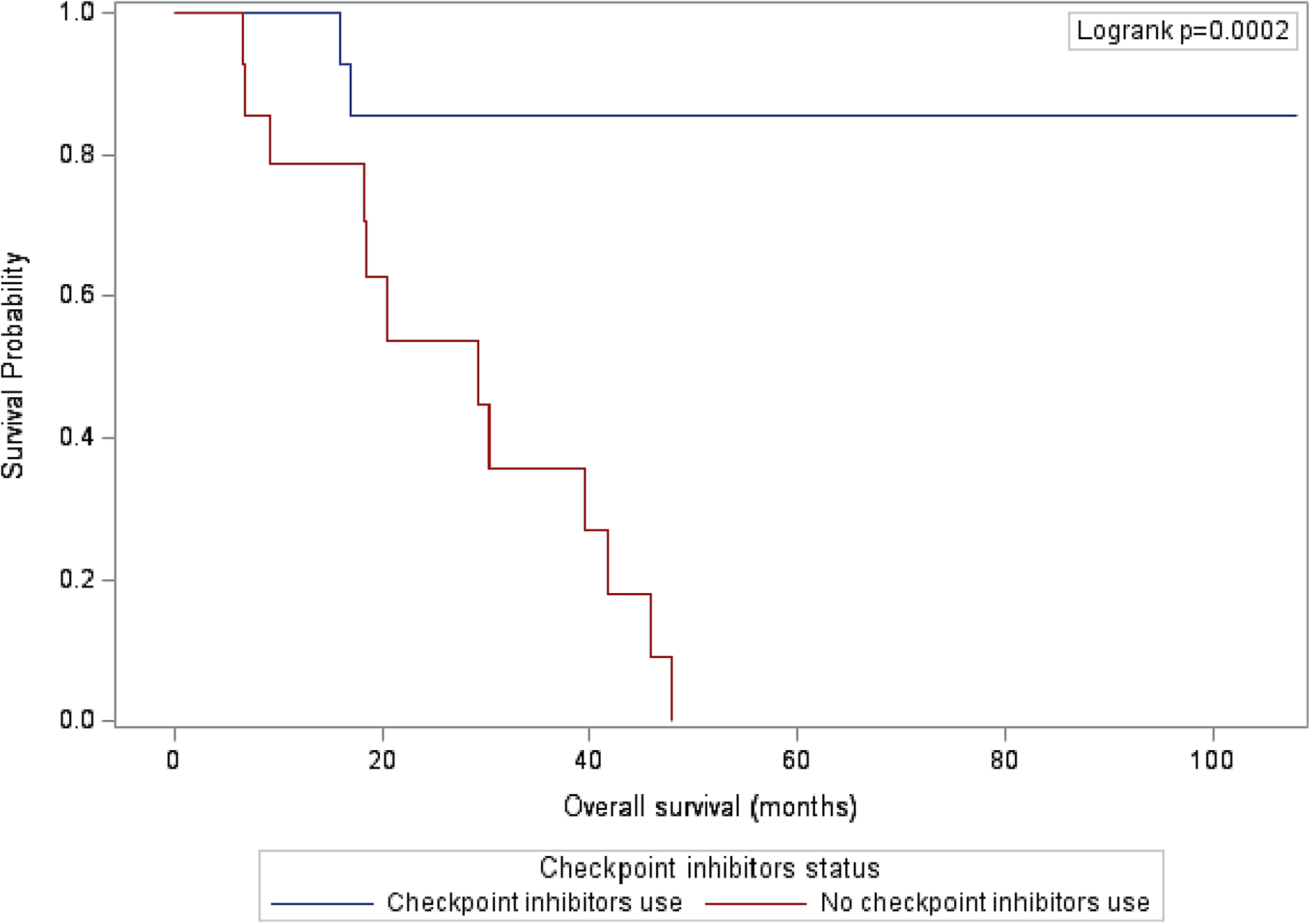
Kaplan–Meier survival estimates for overall survival.

**FIGURE 2:**
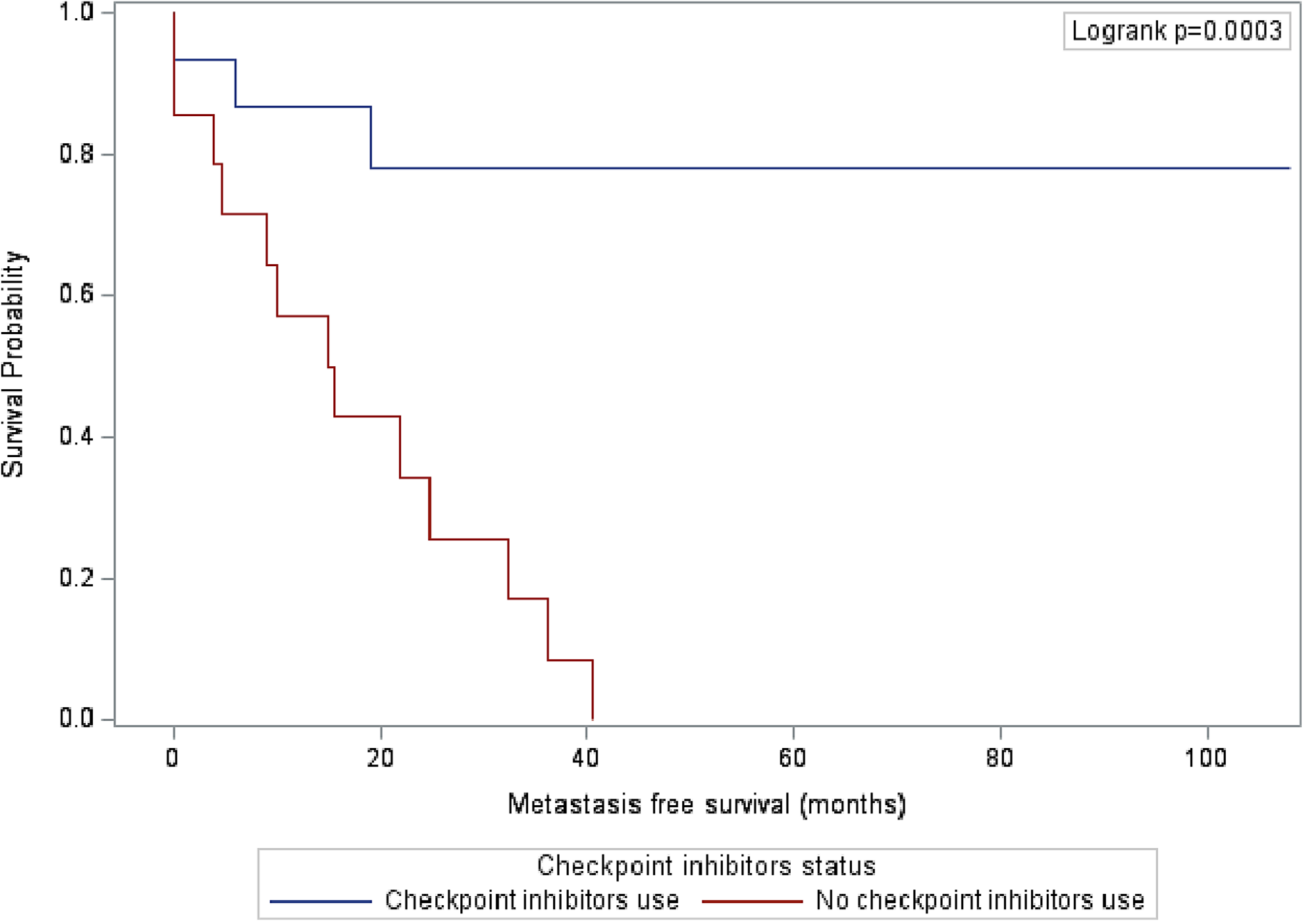
Kaplan–Meier survival estimates for metastasis‐free survival.

In comparison to group 1, both overall and metastasis‐free survival showed significant improvement in group 2 (*p*‐value = 0.0002 and 0.0003, respectively), as illustrated in Figures 1 and 2. At the time of analysis, the majority of patients in group 2 (86.7%) were still alive.

At 12‐month follow‐up, all patients in group 2 were still alive versus 78.6% of patients in group 1 (95% CI: 57.1–100.0). The metastasis‐free survival rate for group 2 was 86.7% (95% CI: 69.5%–100.0), compared to 57.1% (95% CI: 31.2–83.1) in group 1. At 24‐month follow‐up, the overall survival rate for group 2 was 85.7% (95% CI: 67.4–100.0) compared to 53.9% (95% CI: 26.3–81.5) in group1. Similarly, the metastasis‐free survival rate for group 2 was 78.0% (95% CI: 55.7–100.0) compared to 34.3% (95% CI: 8.9–59.9) in group 1.

At nearly 4 years (47.8 months), the overall survival rate in group 1 was 0% (all patients died) versus 85.7% in group 2. The metastasis‐free survival rate was 0% (all patients had metastatic disease) by 40.6 months in group 1, whereas it was 78.0% in group 2 at the same time point.

In the bivariate Cox regression analysis, the utilisation of checkpoint inhibitors (CPIs) was associated with 91% reduction of all‐cause mortality rates [hazard ratio 0.002 (95% CI): 0.02–0.42]. In the multivariable regression analysis, where demographic and clinical variables (such as, age at diagnosis, sex, concomitant bladder cancer and initial tumour size) were adjusted, the risk of all‐cause mortality was nearly 100% lower in ICI users compared to non‐users.

## 4 DISCUSSION

This study aims to evaluate the role of ICIs in patients with high‐grade UTUC treated endoscopically when NU is not feasible. The study spans over two and a half decades, compiling data from a particular patient cohort where local control of the high‐grade disease is achieved ureteroscopically through a stringent follow‐up regimen. Patients were subsequently categorised based upon utilisation of ICIs into two groups to evaluate the role of ICIs on the overall and metastasis‐free survival outcomes.

The standard management approach for high‐risk localised UTUC involves radical NU with excision of the bladder cuff, often accompanied by perioperative chemotherapy.^8^ In case of metastatic disease, a platinum‐based chemotherapy regimen remains the recommended first‐line treatment option.^8^ There has been growing evidence supporting the effectiveness of ICIs in the treatment of urothelial carcinoma, leading to the approval of several agents for use as first‐ and second‐line therapy for advanced urothelial carcinoma.^9^ However, patients with UTUC constitute a minority in clinical trial populations due to the relative rarity of the disease. Furthermore, robust data demonstrating the benefits of ICI utilisation in the context of high‐grade UTUC are limited. We hypothesised that adding systemic treatment using ICIs with local control measures would enhance overall survival for UTUC, given the similarities in histopathological patterns with bladder carcinoma.

The present study groups were comparable regarding patient demographics and disease characteristics. Our approach for ureteroscopic treatment of UTUC employs the ‘no‐touch’ technique, in which the flexible ureteroscope is passed directly into the ureteral orifice under direct vision. This technique minimises access trauma, which could otherwise obscure a malignant lesion or lead to a false positive result.^10^, ^11^ A majority of patients (44%) had tumours larger than 3 cm, and approximately 58.6% underwent staged ureteroscopic treatment. This is largely attributed to the high‐grade nature of the tumours within the study cohort.

The introduction of ICIs has revolutionised the management armamentarium of urothelial carcinoma. Various anti‐programmed cell death (PD)‐1/ligand‐1 (PD‐L1) agents have been approved for locally advanced or metastatic UC following platinum‐based treatment. Notably, pembrolizumab is the only therapy that has been approved by both the FDA and European Medicines Agency based on level 1 evidence. Additionally, pembrolizumab was approved as first‐line treatment in cisplatin‐ineligible patients with high PD‐L1 expression.^6^, ^12^, ^13^

Similarly, in our study, pembrolizumab was the preferred choice as a first‐line treatment in group 2. Atezolizumab was also administered to a minority of patients. The selection of pembrolizumab is supported by the published evidence of its safety and effectiveness in the context of non‐metastatic muscle‐invasive bladder carcinoma. The PURE‐01 trial, for instance, demonstrated the safety and efficacy of short courses of neoadjuvant pembrolizumab prior to radical cystectomy, with 42% of patients achieving pT0 and 54% with <T2 responses. While a few immune‐related adverse events were reported, they did not lead to any delays in planned surgery.^14^

Subsequently, the PURE‐02 study was carried out as a feasibility study of neoadjuvant pembrolizumab involving 10 patients with non‐metastatic high‐risk features UTUC.^15^ The treatment protocol comprised three courses of 200 mg intravenous pembrolizumab administered every 3 weeks, followed by NU within 14 days of the last dose. Mortality was recorded in one patient due to pneumonia and sepsis within a month of receiving the first pembrolizumab course. Additionally, a complete clinical and radiological response was reported in one patient who later declined to undergo NU. Two patients experienced disease progression, necessitating subsequent cisplatin‐based chemotherapy. The remaining cohort (70%) demonstrated uncertain responses or clear non‐responses to the neoadjuvant treatment. Notably, in addition to the small sample size, the authors attributed the conflicting results observed in PURE‐01 and PURE‐02 to the inherent biological diversity of tumours based on the primary location (bladder vs upper tract) along the urothelium. This remains an area of ongoing investigations.^16^, ^17^

There has been growing evidence on the differences in tumour biology and immune microenvironment between UTUC and bladder carcinoma. A recent review showed that the genomic and transcriptomic profiles of high‐grade UTUC indicates a notable prevalence of FGFR3 (fibroblast growth factor receptors) mutations. Additionally, UTUC tends to exhibit the luminal papillary subtype, characterised by a relatively lower presence of immune cells, particularly T cells. These observations collectively imply that UTUC may exhibit a reduced response to immunotherapy when compared to bladder carcinoma.^18^ The present study results showed significantly improved survival outcomes in group 2, which can now serve as an evidence of the effectiveness of ICIs in high‐grade UTUC.

The reliability of PD‐L1 expression in UTUC as a predictor of immunotherapy efficacy is currently under investigation. Larger sample sizes are needed to validate its positive correlation.^19^ A recent study showed that immunohistochemistry of urine cell blocks is a dependable method for assessing PD‐L1 levels, with a 10% cut‐off appearing to be the optimal threshold for predicting the effectiveness of ICIs in UTUC patients.^20^

In our study, erdafitinib was also employed as a second‐line treatment. Erdafitinib is a pan‐fibroblast growth factor (FGF) tyrosine kinase inhibitor approved for locally advanced or metastatic UC with FGFR mutations. In an open‐label phase II study involving 99 patients (including 23 with UTUC) who had locally advanced, unresectable or metastatic urothelial carcinoma, the use of erdafitinib was associated with a 40% response rate. Interestingly, patients who had previously received immunotherapy showed an even higher response rate of 59%.^21^ Of note, additional FGFR‐directed agents like infigratinib and rogaratinib are still under investigation, as well as trials that explore combining these agents with ICIs and chemotherapy regimens.

In the present study, adverse reactions associated with ICIs and related to the immune system were noted in 27% of patients. In most cases, a short course of glucocorticoids, such as prednisolone, is employed as the primary immunosuppressive therapy. If steroid treatment proves ineffective, additional agents like infliximab, adalimumab, golimumab, etanercept, mycophenolate and mofetil can be considered.^22^ Concerns regarding the potential reduction of the anti‐tumour efficacy of ICI therapy due to immunosuppression have been addressed. Retrospective studies have indicated that clinical outcomes remain comparable between patients who receive immunosuppression to manage immune‐related adverse events and those who do not receive such treatment.^23^, ^24^

To the best of our knowledge, our study represents the first report demonstrating the survival benefits of ICIs in non‐surgically treated patients with high‐grade UTUC; however, it is not without limitations. First, the non‐randomised design of the study introduces inherent selection bias. The second limitation is the relatively small sample size from a single centre, which may affect the generalisability and reproducibility of the results. However, given the rarity of the disease and the specific criteria of the patient cohort, conducting a randomised trial with a larger sample size could pose challenges. Lastly, the extended time span of the study introduces potential confounding variables, including improvements in overall survival that may have occurred over this extended duration. Nevertheless, we believe that our results can be a framework for larger multicentre studies to provide more robust evidence regarding the efficacy of ICIs in this specific patient cohort.

## 5 CONCLUSION

Utilisation of adjuvant ICIs in the setting of endoscopically treated patients with high‐grade UTUC is associated with significantly improved overall and metastatic free survival rates. ICI therapy is not without risks and should be administered with caution. Further assessment of its efficacy in treating high‐grade UTUC should be pursued through well‐designed randomised trials.

## AUTHOR CONTRIBUTIONS

Mahmoud Khalil made significant contributions to data collection, interpretation and drafting and editing of the original manuscript. Andrew Fishman contributed significantly to the study design, data collection and manuscript revision. Anna Komorowski was significantly involved in the study design and data interpretation, and provided insights into substantial parts of the manuscript. Israel Franco performed the formal data analysis and interpretation. Michael Grasso contributed to data acquisition and interpretation, as well as reviewing and editing the manuscript. All the authors meet criteria of authorship, have read the manuscript, and have approved this submission.

## CONFLICT OF INTEREST STATEMENT

The authors disclose no potential conflict of interest.
